# The influence of concern about COVID-19 on mental health in the Republic of Georgia: a cross-sectional study

**DOI:** 10.1186/s12992-020-00641-9

**Published:** 2020-11-18

**Authors:** Nino Makhashvili, Jana Darejan Javakhishvili, Lela Sturua, Ketevan Pilauri, Daniela C. Fuhr, Bayard Roberts

**Affiliations:** 1 Mental Health Resource Centre, Ilia State University, Q. Cholokashvili Av. 3/5. E 122., Tbilisi, Georgia; 2 Institute of Addictions, Ilia State University, Q. Cholokashvili Av. 3/5. E 122., Tbilisi, Georgia; 3 National Center for Disease Control and Public Health of Georgia, 99, Kakhety highway, P. Shotadze Tbilisi Medical Academy, 51/2 Ketevan Dedofali Ave, Tbilisi, Georgia; 4 Ilia State University, Q. Cholokashvili Av. 3/5. E 122., Tbilisi, Georgia; 5 Department of Health Services Research and Policy, London School of Hygiene and Tropical Medicine, 15-17 Tavistock Place, London, UK

**Keywords:** COVID-19, Georgia, Mental health

## Abstract

**Background:**

Early evidence indicates increased mental health burden arising from COVID-19 and related control measures. The study aim was to examine concern about COVID-19 and its association with symptoms of mental disorders in the Republic of Georgia.

A cross-sectional internet-based survey of adults in Georgia using non-probabilistic sampling was used. Questionnaire topics were: (i) demographic and socio-economic characteristics; (ii) level of burden caused by common causes of COVID-19 related concerns; (iii) strategies used in response to concerns about COVID-19; and (iv) symptoms of mental disorders of anxiety (GAD-7), depression (PHQ-9), PTSD (ITQ) and adjustment disorder (ADNM8). Descriptive and multivariate analyses were conducted.

**Results:**

There were 2088 respondents. High levels of symptoms for mental disorders were observed for anxiety (23.9% women, 21.0% men), depression (30.3% women, 25.27% men), PTSD (11.8% women, and 12.5% men), and adjustment disorder (40.7% women, 31.0% men). Factors significantly associated with increased COVID-19 concern included bad/very bad household economic situation, larger household size, current NCD, symptoms of anxiety, adjustment disorder and PTSD. Response strategies significantly associated with reduced mental disorder symptoms included meditation and relaxation exercises, physical exercise, positive thinking, planning for the future, TV/radio, housework/DIY, and working. Drinking alcohol was associated with a greater probability of increased mental disorder symptoms.

**Conclusions:**

High levels of mental disorders were recorded, and they were strongly associated with increased concern about COVID-19. A number of response strategies were identified which may help protect against worse mental health and these could be supported by innovations in mental health care in Georgia.

## Introduction

Rapid public health responses to pandemics such as COVID-19 involving physical distancing and other infection prevention and control measures are effective in reducing risk of exposure or infection and thereby avoiding deaths and disability [1]. However, early reports from China on COVID-19 also indicate that they may increase the mental health burden in the location where lockdown is enforced [2, 3]. A recent meta-analysis on mental health and COVID-19 among the general population in China estimates the prevalence of anxiety to be around 31.9%, and depression to be around 33.7% respectively [3]. It is assumed that those symptoms of mental disorders are partly caused by the imposed physical distancing measures leading to social isolation and a break-down of social networks [4, 5]. These findings confirm those of earlier infectious disease outbreaks such as for Ebola, Swine flu and SARS in which increased psychological distress including symptoms of post-traumatic stress disorders (PTSD), depression and anxiety were reported as well [6–8]. The economic downturn and reduced earnings following lockdown might further exacerbate psychological distress, and increase vulnerability of populations with lower socio-economic status; and similarly for those with pre-existing physical illnesses [4, 5, 9–11]. A disproportionate mental health effect from COVID-19 may also be seen in people who already have a mental disorder, and this may lead to relapse and worsening of prognosis if disruptions in their mental health care are experienced [11, 12]. Other outcomes such as symptoms of PTSD and adjustment disorders might also be occurring in people who have traumatic experiences related to COVID-19 including the deaths of family or friends, or the inability to care for sick family members [12]. More direct neuropsychiatric sequalae of COVID-19 on mental health are expected as well [13]. Recent evidence shows that coronaviruses may also affect the central nervous system leading to a significant psychiatric and neuro-psychiatric burden among infected individuals in the acute or post-illness stage [7, 13]. This may include neuro-psychiatric conditions such as confusion, impaired memory, or insomnia in addition to heightened PTSD, depression and anxiety in individuals who have survived severe illness [7, 8]. It is important to better understand the how COVID-19 and related control measures are influencing mental health and what coping strategies individuals utilise in response [5].

Georgia, a country located at the intersection of Europe and Asia, took early public health measures to limit the spread of the virus (details are provided in Additional file 1: Online Annex 1) [14]. This included suspension of flights from major hotspots such as China and Iran in the early months of 2020, and a declaration of a public health emergency in March 2020 which triggered the closure of schools and universities, cancellation of cultural and sport events, and the shutdown of non-essential businesses including restaurants and bars [15]. Gatherings of more than three people were prohibited, and movement restrictions were enforced by erecting checkpoints in Tbilisi and other larger cities in the country [14]. This led to suppression of the virus when case numbers were still manageable; at the end of our survey, Georgia reported around 1000 cases of COVID-19, and 15 deaths [16]. Due to high demand from the population, a national mental health hotline was introduced. However, the impact of COVID-19 on population’s mental health may still be profound [17, 18]. Georgia has a high burden of neuropsychiatric disorders, amounting to 22.8% of the burden of disease pre-COVID-19 [19]. The mental health system of Georgia is under reform but the treatment gap is still wide [20, 21]. The burden of mental disorders and treatment gap may further worsen in the context of COVID-19. The wider research community and the Inter-Agency Standing Committee of the United Nations have called for immediate monitoring to assess the mental health pandemic across countries and more vulnerable populations groups [9, 11, 22]. This may aid understanding of mechanisms of impact and support detecting factors which may be modifiable, in order to help enable secondary prevention and recovery [9].

The aim of this study is to examine concern about COVID-19 and its association with symptoms of mental disorders in the Republic of Georgia. The specific objectives are to examine: (i) the frequency and perceived burden of COVID-19 related concerns; (ii) how concerns about COVID-19 may influence levels of mental disorder symptoms; (iii) what factors are associated with these concerns; and (iv) what individual coping strategies are used to respond to these concerns and how they may influence levels of mental disorder symptoms.

## Methods

A cross-sectional internet-based survey design was used. The population of interest was the general population in Georgia aged 18 years and above. A non-probabilistic sampling design was used. While random sampling would clearly have been preferable, it was not possible to obtain mobile phone records or other types of data required for the sampling frame during the lockdown period in Georgia. The use of a non-probabilistic sample means the data are not nationally representative and do not provide reliable prevalence data. However, basic descriptive analyses and explorations of potential associations can be appropriate in such surveys [23] and were used in the majority of previous surveys on mental health and COVID-19 which were conducted so far [24]. For the recruitment, survey weblinks were shared through social and traditional media, key health agencies in Georgia, and investigator networks (see Additional file 2: Online Annex 2 for further details on the recruitment strategy).

The study questionnaire consisted of questions on: (i) background demographic and socio-economic characteristics (e.g. age, sex, education, employment, income-level, socio-economic status); (ii) 19 questions on the level of burden caused by common causes of COVID-19 related concerns over the previous 1 month (developed through consultation and piloting - see results section for items); (iii) individual coping strategies used in response to concerns about COVID-19 (adapted from COPE and through piloting – see results section for items) with a recall period of since the beginning of the COVID-19 crisis in Georgia (February 2020); and (iv) symptoms of mental disorders of anxiety, depression, PTSD and adjustment disorder. Anxiety was measured using the Generalised Anxiety Disorder (GAD-7) instrument which consists of 7 questions on anxiety symptoms over the last 2 weeks, with the same response options and scoring as the PHQ-9 which produces a total score range of 0–21, with the GAD-7’s suggested cut-off of ≥10 used to indicate symptoms of at least moderate anxiety [25]. Depression was measured using the Patient Health Questionnaire (PHQ-9) which consists of 9 questions on depression symptoms over the last 2 weeks, with responses of ‘not at all’ (=0), ‘several days’ (=1), ‘more than half the days’ (=2), and ‘nearly every day’ (=3), with item scores summed to produce a total score range of 0–27, with the PHQ-9’s suggested cut-off of ≥10 used to indicate symptoms of at least moderate depression [26]. PTSD was measured using an adapted version of the International Trauma Questionnaire (ITQ) with respondents asked 8 questions about emotional reactions to COVID-19, covering symptom clusters of re- experiencing in the here and now, avoidance, sense of current threat; and functional impairment associated with these symptoms. Response options ranged from ‘not at all’ to ‘extremely’; and overall symptoms of PTSD required endorsement of one of two symptoms from the three symptom clusters plus endorsement of at least one indicator of functional impairment [27]. Adjustment disorder was measured using an adapted version of the Adjustment Disorder – New Module 8 (ADNM 8), with 8 questions asked about emotional reactions to COVID-19, with response options of 1 to 4 for each questions and a total score of 8 to 32 covering two main clusters of preoccupation related to the stressor and failure to adapt to the stressor, with a cut-off used of 18.5 as recommended elsewhere [28, 29]. The mental health outcome measures were selected as they have been widely used in a number of settings, including in Georgia [30, 31]. They also showed good internal reliability, with Cronbach alpha scores ranging from 0.89 to 0.91 for the four measures. Results on tests on construct validity were also good. For example, tests on factor analysis showed single factors for each of GAD-7, PHQ-9 and ITQ; while ADNM-8 showed two factors corresponding with the two dimensions around pre-occupation with stressor and failure to adapt.

The questionnaire was first developed in English between the co-investigators and shared with other mental health experts in Georgia for review. It was then translated into Georgian language using standard questionnaire translation methods [32], and piloted with 19 participants to examine feasibility, clarity, errors, and ethical issues. It was hosted on the Jisc online survey platform (https://www.jisc.ac.uk) and launched on 25 May 2020 and closed on 25 June 2020.

Data analysis was conducted using Stata 16 (StataCorp, College Station, TX, USA). It included descriptive analysis of the study sample, levels of symptoms of mental disorders, the frequency and burden of concern about COVID-19, and strategies used by respondents in response to COVID-19 related concerns.

Multivariate regression analysis was conducted for two main purposes. First, to examine the factors associated with an outcome of greater burden of concern about COVID-19. The dependent variable of greater burden of concern about COVID-19 was developed by totalling the scores from the 19 questions on the level of burden from individual COVID-19 concern items which each had 4 response options ranging from ‘no concern at all’ (=1) to ‘strongly burdened’ (=4), resulting in a score range from 19 to 76. The independent variables were selected by initial testing through univariate analysis and those showing a significant association with the dependent variable (*P* < 0.05) were entered into the full model and backward stepwise regression analysis was then used to select the final significant variables (P < 0.05). The second use of regression analysis was to examine associations between response strategies to concern about COVID-19 (independent variables) with symptoms of mental disorders (‘dependent variable). Separate multivariate models were run for each response strategy and the different mental disorders using the cut-offs noted above, adjusting for gender, age, education, household economic status, household size, and having an existing mental disorder.

Ethics approval was provided by the National Centre for Disease Control and Public Health in Georgia.

## Results

There were 2088 respondents and the sample characteristics are provided in Table 1. Women comprised 1807 (86.5%) of the study sample. 23.4% of women and 19.2% of men were aged over 50 years. Over two-thirds of the study sample lived in Tbilisi and one-fifth considered their household situation was bad or very bad (with no significant difference between women and men). High levels of symptoms for mental disorders were observed for anxiety (23.9% women, 21.0% men), depression (30.3% women, 25.27% men), PTSD (11.8% women, and 12.5% men), and adjustment disorder (40.7% women, 31.0% men). 14.8% of women and 15.6% of men in the study sample had previously been diagnosed with a mental disorder. Of note, of the 953 respondents who had symptoms of any mental disorder in our study, 23.2% had previously been diagnosed with a mental disorder. Further data on mental disorder symptom scores are provided in Additional file 3: Online Annex 3, and Additional file 4: Online Annex 4 provides data separated out by previous mental health diagnosis status.

**Table 1:** Sample Characteristics, by gender (*N* = 2088)

	Women		Men	
	N	%	N	%
**Total**	1807	86.54	281	13.46
**Age groups:**				
18–39	996	55.12	182	64.77
40–49	388	21.47	45	16.01
50–59	300	16.60	27	9.61
60–69	111	6.14	22	7.83
70 and over	12	0.66	5	1.78
**Education:**				
Incomplete secondary	27	1.49	8	2.85
Completed secondary	98	5.42	18	6.41
Incomplete higher education	300	16.6	46	16.37
Completed higher education	1382	76.48	209	74.38
**Living location:**				
Tbilisi	1255	69.45	193	68.68
Regional Centre	398	22.03	66	23.49
Village	154	8.52	22	7.83
**Household economic situation:**				
Very good	26	1.45	12	4.27
Good	319	17.81	50	17.79
Average	1115	62.26	160	56.94
Bad	263	14.68	50	17.79
Very bad	68	3.80	9	3.20
**Symptoms of mental disorders:**^**a**^				
Anxiety	432	23.91	59	21.00
Depression	548	30.33	71	25.27
PTSD	213	11.79	35	12.46
Adjustment Disorder	736	40.73	87	30.96^b^
Previously diagnosed with a mental disorder	260	14.81	42	15.61
**Current NCDs:**				
Diagnosed diabetes	42	2.32	10	3.56
Diagnosed hypertension	146	8.08	23	8.19
Diagnosed cardiovascular disease	53	2.93	12	4.27
Diagnosed cancer	20	1.11	2	0.71
Respiratory illness	66	4.00	12	4.27

^a^GAD-7 anxiety score > 9; PHQ-9 depression score > 9; ADNM 8 adjustment disorder score > 18.4; see methods section for PTSD (ITQ) scoring and further details

^b^Statistically significant difference between women and men at *P* < 0.05 (Chi2)

Respondents were also asked about changes in their smoking and alcohol use since COVID-19 lockdown measures were introduced (data not shown in tables). For smoking, 34.6% of women and 19.9% men reported increased rates of smoking, whereas 18.8% women and 18.4% men reported decreased rates (with the remaining respondents reporting about the same levels of use). In contrast, 13.5% women and 12.2% men reported more alcohol use since lock-down measures were introduced, while 35.6% women and 38.6% men reported reduced use of alcohol (with the remaining respondents reporting about the same levels of use).

The findings on the major causes of concern related to COVID-19 are provided in Table 2. The most frequent major causes of concern were related to infecting loved ones and others with COVID-19, uncertainty about duration and risks of COVID-19, income loss, restricted contract with family and friends, being socially isolated, being at home, and restrictions on daily activities.

**Table 2:** Number of respondents feeling ‘strongly burdened’ by individual concerns related to COVID-19 (*N* = 2088)

	Women (***N*** = 1807)		Men (***N*** = 281)	
	N	%	N	%
Loved ones get infected with C19	1116	62.17	149	53.41^a^
Infecting others with C19	833	48.86	117	44.15
Uncertainty about duration and risks of C19 pandemic	835	46.41	99	35.61^a^
Income loss	663	44.26	96	39.67
Restricted contact with family, friends etc	779	43.45	119	44.40
Being socially isolated	628	35.42	87	32.10
Being at home	616	35.59	87	34.12
Restricted everyday activities	505	28.32	81	29.67
No place of retreat	430	27.78	60	24.79
Insufficient capacity of intensive care	355	26.49	51	22.37
Becoming infected	457	25.33	42	15.00^a^
Restricted home space	359	22.52	51	20.65
Childcare	194	18.87	20	12.27^a^
Poor information from authorities	303	17.23	45	16.61
Home working	202	16.96	24	12.57
Restricted access to food and essential goods	239	13.73	34	12.55
Conflict at home	159	12.10	22	11.06
Access to routine healthcare	204	11.99	28	10.73
Violence at home	34	3.66	8	5.30

Denominator reduced in some responses as question may not be applicable to all (e.g. childcare)

^a^Statistically significant difference between women and men at P < 0.05 (Chi2)

The mean COVID concern score was 43.15 [95% CI 42.69 to 43.60] out of a potential range of 19 to 76. For women the mean score was 43.37 [95% CI 42.88 to 43.86] and for men it was 41.71 [95% CI 40.45 to 42.97). The factors associated with increased concern about COVID-19 identified through the multivariate regression analysis are shown in Table 3. A bad or very bad household economic situation showed a significant association with greater COVID-19 concern (Coef. 2.66 [95% CI 1.36 to 3.96) (i.e. it was associated with a 2.66 change in the mean score of concern about COVID-19), as did living in a larger household size (rising to Coef. 4.58 [95% 2.99 to 6.16]). Having a current non-communicable disease (NCD) was associated with greater concern (Coef. 1.28 [95% 0.08 to 2.49]). Having symptoms of mental disorders was strongly associated with greater concern about COVID-19, with anxiety the strongest (Coef. 5.62 [95% 4.45 to 6.79]), then adjustment disorder (Coef. 4.57 [95% 3.60 to 5.55]), and then PTSD (Coef. 2.78 [95% 1.40 to 4.16]). Neither depression nor prior diagnosis with a mental disorder showed a statistically significant association in the multivariate model. The factors associated with less concern about COVID-19 were increasing age (but not for those aged 70 and above) and being in a higher-risk occupation (Coef. -1.65 [95% -2.69 to − 0.60]) such as a health worker, social worker, transport driver, or essential shop worker.

**Table 3:** Factors associated with concern about COVID-19 (*N* = 2058)

	Coef.	***P***	[95% Conf. Interval]	
**Age:**				
18–39	Ref			
40–49	−0.50	0.33	[−1.51;	0.50]
50–59	−2.80	< 0.01	[−3.97;	−1.62]
60–69	−4.14	< 0.01	[−5.87;	−2.42]
70 and over	−1.23	0.59	[−5.70;	3.25]
**Household economic situation:**				
Very good or good	Ref			
Average	0.07	0.89	[−0.94;	1.08]
Bad or very bad	2.66	< 0.01	[1.36;	3.96]
**Household size:**				
1 person	Ref			
2 persons	1.12	0.22	[−0.66;	2.90]
3 persons	3.67	< 0.01	[2.02;	5.31]
4 persons	3.16	< 0.01	[1.54;	4.79]
5 or more persons	4.58	< 0.01	[2.99;	6.16]
**General health status:**				
Very good	Ref			
Good	2.59	< 0.01	[1.29;	3.88]
Average	3.61	< 0.01	[2.21;	5.00]
Bad	1.64	0.15	[−0.57;	3.86]
Very bad	3.17	0.28	[−2.59;	8.93]
**Higher risk occupation:**				
No	Ref			
Yes	−1.65	< 0.01	[− 2.69;	−0.60]
**Current NCDs:**				
No NCD	Ref			
Any NCD^a^	1.28	0.04	[0.08;	2.49]
**Symptoms of mental disorders:** ^**b**^				
Anxiety	5.62	< 0.01	[4.45;	6.79]
Adjustment disorder	4.57	< 0.01	[3.60;	5.55]
PTSD	2.78	< 0.01	[1.40;	4.16]

Results are adjusted for other variables in table using backward stepwise multivariate regression (R-Squared = 0.30)

^a^One or more of diabetes, hypertension, CVD, or cancer

^b^GAD-7 anxiety score > 9; PHQ-9 depression score > 9; ADNM 8 adjustment disorder score > 18.4; see methods section for PTSD (ITQ) scoring. Reference category is no mental disorders

The individual coping strategies used by respondents to help address their concerns about COVID-19 are shown in Table 4. The most common strategies were: seeking emotional support by speaking with family and friends (55.6% women, 43.42% men); reading, watching TV or listening to the radio (54.4% women, 60.1% men); doing housework or DIY (46.2% women, 36.6% men); working (36.9% women, 43.4% men); using social media (36.6% women, 45.9% men); and taking exercise (35.4% women, and 39.2% men).

**Table 4:** Frequencies of response strategies used to address concern about COVID-19 (by gender), and their association with mental health disorder symptoms

Response strategy	Frequencies (N = 2088)				Association with mental health disorder symptoms (***N*** = 2011) ^b^							
	Women		Men		Anxiety ^c^		Depression^c^		PTSD^c^		Adjustment disorder^c^	
	N	%	N	%	OR	*P*	OR	*P*	OR	*P*	OR	*P*
**Emotional support and expression:**												
Emotional support by speaking with family/friends	1005	55.62	122	43.42^a^	**0.71**	***< 0.01***	0.88	*0.25*	0.80	*0.13*	1.06	*0.55*
Going to church/praying	244	13.50	26	9.25^a^	0.77	*0.16*	0.77	*0.12*	0.99	*0.96*	**0.73**	***0.04***
Expressing my negative feelings /crying/arguing/being aggressive	236	13.06	20	7.12^a^	**4.42**	***< 0.01***	**4.45**	***< 0.01***	**4.42**	***< 0.01***	**4.14**	***< 0.01***
**Positive thinking and helping others:**												
Looking for something good in what is happening	591	32.71	85	30.25	**0.41**	***< 0.01***	**0.60**	***< 0.01***	**0.63**	***0.01***	**0.63**	***< 0.01***
Planning for the future, considering facts of changed reality	576	31.88	109	38.79^a^	**0.63**	***< 0.01***	**0.73**	***0.01***	**0.59**	***0.00***	0.84	*0.08*
Helping others	530	29.33	81	28.83	0.79	*0.07*	0.81	*0.07*	0.96	*0.79*	**0.79**	***0.03***
**Exercise and relaxation techniques:**												
Taking exercise	640	35.42	110	39.15	**0.53**	***< 0.01***	**0.55**	***< 0.01***	**0.66**	***0.01***	**0.66**	***< 0.01***
Meditation/relaxation exercises	200	11.07	26	9.25	**0.39**	***< 0.01***	**0.47**	***< 0.01***	**0.39**	***< 0.01***	**0.46**	***< 0.01***
**Professional help & medications:**												
Self-medication on prescribed drugs	94	5.20	10	3.56	**2.71**	***< 0.01***	**3.35**	***< 0.01***	**3.60**	***< 0.01***	**3.21**	***< 0.01***
Getting psychological support (e.g. online counselling/therapy)	90	4.98	12	4.27	**1.92**	***0.01***	**2.33**	***< 0.01***	**3.29**	***< 0.01***	**1.84**	***0.01***
Calling NCDC hotline or family doctor	19	1.05	5	1.78	**2.60**	***0.04***	1.48	*0.41*	1.35	*0.64*	1.25	*0.62*
**Distraction:**												
Reading, TV, radio	983	54.40	169	60.14	**0.52**	***< 0.01***	**0.67**	***< 0.01***	**0.55**	***0.00***	0.85	*0.10*
Doing housework or DIY	835	46.21	103	36.65^a^	**0.63**	***< 0.01***	**0.73**	***< 0.01***	**0.68**	***0.01***	**0.73**	***< 0.01***
Working	666	36.86	122	43.42	**0.74**	***0.02***	**0.62**	***< 0.01***	0.80	*0.20*	0.87	*0.18*
Using social media	661	36.58	129	45.91^a^	0.91	*0.42*	1.21	*0.08*	1.01	*0.93*	1.14	*0.20*
Daydreaming/sleeping	542	29.99	86	30.60	**1.62**	***< 0.01***	**2.33**	***< 0.01***	**1.68**	***< 0.01***	**2.14**	***< 0.01***
Playing a lot of video games	49	2.71	48	17.08^a^	1.28	*0.34*	1.41	*1.97*	1.31	*0.38*	1.25	*0.35*
**Substance use or gambling:**												
Drinking alcohol	149	8.25	55	19.57^a^	**1.50**	***0.03***	**1.97**	***< 0.01***	**1.81**	***0.01***	**1.72**	***< 0.01***
Gambling	13	0.72	11	3.91^a^	1.84	*0.22*	1.55	*0.37*	2.07	*0.19*	1.45	*0.42*
Taking illegal drugs	12	0.66	14	4.98^a^	1.15	*0.78*	0.94	*0.91*	–	*–*	0.81	*0.63*

^a^Frequencies: statistically significant difference between women and men at P < 0.05 (Chi2 test)

^b^Separate multivariate regression models run for association between individual mental disorder and individual response strategy, adjusting for gender, age, education, household economic status, household size, and existing mental health disorder (for brevity, only data for response strategy are shown). Statistically significant (*P* < 0.05) results shown in bold

^c^GAD-7 anxiety score > 9; PHQ-9 depression score > 9; ADNM 8 adjustment disorder score > 18.4; see methods section for PTSD (ITQ) scoring

The results from the multivariate analysis on the associations between the COVID-19 individual coping response strategies used by respondents and symptoms of mental disorders are shown in Table 4 (for brevity, only data for response strategies are shown rather than the other independent variables included in the models), and significant (*P* < 0.05) results are as follows. Meditating and using relaxation exercises were associated with lower symptoms of anxiety (adjusted odds ratio (OR) 0.39 (i.e. representing a 61% decrease in the probability of having symptoms of anxiety after adjustment for other variables in the model]), depression (OR 0.47), PTSD (OR 0.39), and adjustment disorder (OR 0.46). Similarly, use of exercise was associated with lower symptoms of anxiety (OR 0.53), depression (OR 0.55), PTSD (OR 0.66), and adjustment disorder (OR 0.66). Positive thinking was also associated with lower symptoms of anxiety (OR 0.41), depression (OR 0.60), PTSD (OR 0.63), and adjustment disorder (OR 0.63). Trying to plan for the future was associated with lower symptoms of anxiety (OR 0.63), depression (OR 0.73), and PTSD (OR 0.59), but not with adjustment disorder. Distraction techniques of reading/TV/radio, doing housework or DIY, and working were associated with lower symptoms of anxiety (with ORs ranging from 0.52 to 0.74), depression (ORs ranging from 0.62 to 0.73), PTSD (ORs 0.55 to 0.68), and adjustment disorder (ORs 0.73 for doing housework/DIY). Seeking emotional support was associated with lower symptoms of anxiety (OR 0.71) only.

Drinking alcohol was associated with higher symptoms of anxiety (OR 1.50); depression (OR 1.97), PTSD (OR 1.81), and adjustment disorder (OR 1.72). Other factors showing associations with higher symptoms of mental disorders, included expressing negative feelings such as crying, arguing, being aggressive (ORs ranging from 4.14 to 4.45), daydreaming/sleeping (ORs ranging from 1.62 to 2.33), self-medication on prescribed drugs (ORs from 2.71 to 3.60), and getting psychological support such as online counselling or therapy (ORs 1.84 to 3.29).

## Discussion

This is the first study in Georgia which reports on the mental health influence of COVID-19. We found high levels of symptoms of anxiety, depression, PTSD and adjustment disorder. Respondents living in poorer economic circumstances, in multiple households and those with pre-existing physical health (NCDs) and mental health conditions had greater concern about COVID-19. Respondents employed different strategies to cope with COVID-19 concerns which included adaptative and maladaptive approaches. Coping strategies such as meditation, exercise, staying positive and keeping busy/distracting themselves were associated with reduced probability of symptoms of mental disorders, while alcohol use increased the probability of mental disorders.

Research on the mental health influence of COVID-19 and the social and physical restrictions is still in its infancy [2, 13]; however, a pattern of elevated risk of mental health problems and psychological distress emerges [22]. A cross-sectional online survey with the general population in China on mental health in the context of COVID-19 reports the prevalence of anxiety and depression to be around 32% and 34% respectively [3]. Some studies report women to be at greater risk of worse mental health outcomes in the pandemic [24, 33], a finding which we could not confirm in our study with the exception of Adjustment Disorder. Vulnerable groups in our survey such as persons living in poorer economic circumstances and those with pre-existing physical and mental health problems showed greater concern about COVID-19, and it is likely that those population groups may also suffer from heightened psychological distress during the pandemic [5, 34]. Our findings did not show an association with older age and concern about COVID-19 which contrasts with studies from elsewhere [35], but this may be attributable to the low numbers of respondents aged over 70 in our sample. Social isolation and loneliness are strongly associated with excess mortality and mental disorders across the lifespan suggesting that the older population should not be left out from targeted mental health advice.

Respondents in our survey used different individual coping mechanisms to deal with the stress of COVID-19. Positive coping mechanisms such as exercising body and mind, talking to close friends and family, seeking social support, but also diverting thoughts from the current situation seemed to buffer against negative mental health outcomes, and are reported as an effective tool to reduce psychological distress in other studies [36, 37]. Cognitive and behavioural coping strategies which are adaptive should be further promoted at the national level as simple primary prevention advice. A clear message should be provided that alcohol consumption may be maladaptive, and may worsen mental health, as also supported by our findings. Similarly, tobacco consumption should also be strongly discouraged. It is noteworthy that most respondents reported drinking the same or less amount of alcohol since COVID-19 lockdown measures were introduced. This contrasts with what is being reported in other countries [38, 39], and we think may be attributable to the Georgian cultural habits of drinking in groups from different households and the lock-down measures in Georgia meant opportunities for this pattern of drinking were reduced. However, this does require further examination.

Our results also showed that key workers seem to be less worried about COVID-19 and this finding is rather surprising. While it may be attributable to selection bias, another explanation could be that some key workers who choose to play an active role in pandemic situation are more resilient and have more dynamic ability to adapt successfully in the face of adversity, trauma, or significant threat [40]. However, while such resilience may buffer against stressors, it could cause burn out if adverse working conditions go on for a longer time and this requires further investigation [41]. A recent systematic review evidenced that responding to COVID-19 has the potential to significantly affect the mental health of healthcare workers [42], but health workers may be reluctant to disclose mental health difficulties or seek help for them [43].

### Limitations

The primary limitation is the use of non-probabilistic sampling due to the online survey methodology used. This risks sample selection bias, for example by excluding those without internet access who may have greater needs (such as those with severe mental disorders). Therefore, the findings cannot be interpreted to be prevalence data or nationally representative. However, basic descriptive analyses and explorations of potential associations can be appropriate in such surveys [23], and have been used in the majority of COVID-19 mental health surveys which have been done to date [44]. The key effect of the non-probabilistic sampling was the substantial over-representation of women in the study sample (with 87% of the study sample being women, compared to 53% in census data from Georgia) [45], and this is common with other such online surveys [23]. We have responded to this through presenting the descriptive results separately for women and men, and adjusting for gender in the regression analysis. Other demographic data such as age distribution are more in line with Georgian census data, with the exception of an under-representation of those aged 60–69 (6% difference) and aged 70 and over (11% difference) [45], which is perhaps predictable given the online nature of the survey and likely lower use of the internet use among older people in Georgia. There was also significant over-representation of respondents from Tbilisi compared with census data (39% difference) [45]. We are unable to compare our findings on levels of mental disorders against nationally representative data as no such data exist in Georgia and so a key recommendation is the need to conduct nationally representative random sampling surveys on mental disorders and their drivers in Georgia. A second limitation is the cross-sectional design which means temporal causal relationships cannot be observed, most notably between mental disorder symptoms and concern about COVID-19 and related social distancing measures. Third, the self-reported mental health instruments are used for screening mental health symptoms only, rather than diagnosis. They have also not been formally validated in Georgia but they did show good internal reliability and construct validity with the study populations which are also confirmed in previous studies which we have conducted in Georgia [30, 31]. Finally, due to the online convenience sampling approach, we used a relatively brief questionnaire which precluded more in-depth data collection.

## Conclusions

Our survey in Georgia provides a first snapshot on the mental health situation during COVID-19. Further research on this is recommended to substantiate our findings, most notably by conducting a nationally representative random sampling survey. In the meantime, mental health infrastructure should be boosted in anticipation of higher need for mental health services in the near future. This could be accompanied by innovations such as increased use of online services to help reduce the mental health treatment gap in Georgia. Public health researchers and clinicians also need to be mindful of COVID-19 and related responses exacerbating mental health disparities, and so vulnerable groups should be prioritised. Actions taken now may help prevent and mitigate the negative impact of COVID-19 on mental health in Georgia.

## Supplementary Information

**Additional file 1.** **Online Annex 1.** Timeline of COVID-19 responses in Georgia (year 2020).

**Additional file 2.** **Online Annex 2.** Recruitment Strategy.

**Additional file 3.** **Online Annex 3.** Detailed results for symptoms of mental disorders, by gender and previous mental health diagnosis status.

**Additional file 4.** **Online Annex 4.** Detailed results for symptoms of mental disorders, by previous mental health diagnosis status (*N* = 2024).

## Data Availability

Data can be made available upon request to the corresponding author.
