# Supplementary material for: The influence of concern about COVID-19 on mental health in the Republic of Georgia: a cross-sectional study
Source: Global Health. 2020 Nov 18;16:111. doi: 10.1186/s12992-020-00641-9 (PMC7672175; doi:10.1186/s12992-020-00641-9)
Supplement: Supplementary file 1 — Additional file 1. Online Annex 1. Timeline of COVID-19 responses in Georgia (year 2020). [file 12992_2020_641_MOESM1_ESM.docx]

**Online Annex 1: Timeline of COVID-19 responses in Georgia (year 2020)**

| January 29 | Suspension of flights with China |
| --- | --- |
| February 6 | Activation of risk communication; dissemination of educational material and video classes |
| February 12 | Methodological recommendations and protocols developed |
| February 24 | Suspension of air and land traffic with Iran |
| February 26 | First confirmed case in Georgia |
| March 2 | Termination of educational process |
| March 4 | Suspension of air traffic with Italy; Quarantine Zones prepared and personnel trained |
| March 10 | Decentralization of lab diagnostics; social media campaign |
| March 14 | Distant working and educational processes started |
| March 15 | Winter resorts locked down |
| March 18 | Minimization of crossing al the borders; closure of cafe-bars, restaurants, fitness clubs, and pools |
| March 21 | State Emergency declared; inter-city travel suspended |
| March 31 | Total quarantine declared |
| April 3 | Additional restriction imposed under the State of emergency: curfew from 21:00 – 06:00; banned gathering of more than 3 people; suspended travel within and between cities and municipalities. |
| April 8 | Promotion of “Stay Home“ campaign |
| April 13 | Lockdown of a village at Khashuri municipality |
| April 14 | State Emergency prolonged until May 22 |
| April 15 | Suspension of road traffic between Tbilisi, Batumi, Kutaisi and Rustavi |
| May 5 | Intercity driving between Kutaisi and Batumi reopened |
| May 8 | Stringent quarantine measures cancelled in Kobuleti |
| May 11 | Tbilisi reopened; all retail and wholesale stores opened |
| May 18 | Beauty salons opened |
| May 23 | State of Emergency ended and the curfew cancelled; cancellation of restrictions of limit of 3 persons per vehicle |
