# Supplementary material for: The influence of concern about COVID-19 on mental health in the Republic of Georgia: a cross-sectional study
Source: Global Health. 2020 Nov 18;16:111. doi: 10.1186/s12992-020-00641-9 (PMC7672175; doi:10.1186/s12992-020-00641-9)
Supplement: Supplementary file 2 — Additional file 2. Online Annex 2. Recruitment Strategy. [file 12992_2020_641_MOESM2_ESM.docx]

### **Online Annex 2: Recruitment Strategy**

| **Source** | **General population** | **Elderly** | **Frontline  providers** | **Persons with mental disorders** | **Persons with chronic health disorders** | **People living in the conflict zone & displaced** |
| --- | --- | --- | --- | --- | --- | --- |
| Individual Facebook (FB) networks | X |  |  |  |  | X |
| Organizations’ FB pages: NCDC, GIP-T, Iliauni | X |  | X |  | X |  |
| FB Groups: Synergy,MSc students, etc. | X |  |  |  |  |  |
| FB live announcements: | X |  |  |  |  |  |
| Mailing list of Iliauni | X |  |  |  |  |  |
| Mailing list of OSGF | X |  |  |  |  |  |
| Mailing list of WHO-Georgia | X |  |  |  |  |  |
| Mailing list of Georgian Red Cross MHPSS network | X |  | X |  |  |  |
| Mailing list of UNDP | X |  |  | X | X |  |
| Mailing list of GIP-T MH2R Platform, plus 17 NGOs from regions. | X |  |  | X |  | X |
| Mailing list of ‘Alliance for Better MH’ | X |  |  | X |  |  |
| Curatio International mailing list, including professional medical societies (Healthcare Advocacy Coalition) | X |  |  |  |  |  |
| Mailing list of GCRT/Kutaisi | X |  |  |  |  |  |
| Mailing list of Synergy Batumi | X |  |  |  |  |  |
| Mailing list of GCRT/Gori and other IDP organizations | X |  |  |  |  | X |
| Mailing list of mental health service usser organization – O.Kalina | X |  |  | X |  |  |
| Mailing list of Telavi – Alter Ego network | X |  |  | X |  |  |
| Mailing list of Ucha Vaxania SCO/Network working with elderlies | X | X |  |  |  |  |
| Media –central (ghn agency, getgazeti) | X |  |  |  |  |  |
| Media means – local (‘Batumelebi’) | X (Batumi) |  |  |  |  |  |
| Interviews by project staff on TV | X |  |  |  |  |  |
| Announcements disseminated through clinics, apothecaries, supermarkets, barber shops, etc. | X |  |  |  |  |  |
| Mailing list of the Georgian Harm Reduction Network, other drug-focused organizations, and HIV/AIDS Prevention Country Platform, addiction clinics | X |  |  | X | X |  |
| MoH and Parliament of Georgia | X |  |  |  |  | X |
| Social Protection Agency | X | X |  |  | X |  |
| Republican Emergency Clinic | X |  |  |  |  |  |
| NCDC network | X |  |  |  |  |  |
