# Supplementary material for: The influence of concern about COVID-19 on mental health in the Republic of Georgia: a cross-sectional study
Source: Global Health. 2020 Nov 18;16:111. doi: 10.1186/s12992-020-00641-9 (PMC7672175; doi:10.1186/s12992-020-00641-9)
Supplement: Supplementary file 3 — Additional file 3. Online Annex 3. Detailed results for symptoms of mental disorders, by gender and previous mental health diagnosis status. [file 12992_2020_641_MOESM3_ESM.docx]

**Online Annex 3: Detailed results for symptoms of mental disorders, by gender and previous mental health diagnosis status**

| **Mental Disorder** (scoring cut-offs) | **Women (N=1807)** | | | |  | **Men (N=281)** | | | |
| --- | --- | --- | --- | --- | --- | --- | --- | --- | --- |
|  | **N** | **%** | **[95% Conf.** | **Interval]** |  | **N** | **%** | **[95% Conf.** | **Interval]** |
| **Anxiety symptoms (GAD-7):** |  |  |  |  |  |  |  |  |  |
| Minimal (0-4) | 680 | 37.63 | [35.42; | 39.89] |  | 133 | 47.33 | [41.53; | 53.20] |
| Mild (5-9) | 695 | 38.46 | [36.24; | 40.73] |  | 89 | 31.67 | [26.48; | 37.37] |
| Moderate (10-14) | 285 | 15.77 | [14.16; | 17.53] |  | 41 | 14.59 | [10.91; | 19.24] |
| Severe (15-21) | 147 | 8.14 | [6.96; | 9.49] |  | 18 | 6.41 | [4.06; | 9.96] |
| *Mean anxiety score* |  | *6.72* | *[6.49;* | *6.94]* |  |  | *5.88* | *[5.29;* | *6.47]* |
| **Depression (PHQ-9):** |  |  |  |  |  |  |  |  |  |
| Minimal (0-4) | 596 | 32.98 | [30.85; | 35.19] |  | 103 | 36.65 | [31.20; | 42.47] |
| Mild (5-9) | 663 | 36.69 | [34.50; | 38.94] |  | 107 | 38.08 | [32.56; | 43.92] |
| Moderate (10-14) | 308 | 17.04 | [15.38; | 18.85] |  | 39 | 13.88 | [10.29; | 18.46] |
| Moderately severe (15-19) | 153 | 8.47 | [7.27; | 9.84] |  | 21 | 7.47 | [4.91; | 11.21] |
| Severe (20-27) | 87 | 4.81 | [3.92; | 5.90] |  | 11 | 3.91 | [2.18; | 6.95] |
| *Mean depression score* |  | *7.77* | *[7.51;* | *8.03]* |  |  | *7.05* | *[6.38;* | *7.71]* |
| **PTSD (ITQ):** |  |  |  |  |  |  |  |  |  |
| PTSD symptoms* | 213 | 11.79 | [10.30; | 13.28] |  | 35 | 12.46 | [8.57; | 16.34] |
| **Adjustment Disorder (ADNM-8):** |  |  |  |  |  |  |  |  |  |
| Adjustment disorder symptoms (18.5-32) | 736 | 40.73 | [38.46; | 43.00] |  | 87 | 30.96 | [25.81; | 36.63] |
| *Mean adjustment disorder score* |  | *17.80* | *[17.51;* | *18.10]* |  |  | *16.22* | *[15.50;* | *16.95]* |
