# Supplementary material for: The influence of concern about COVID-19 on mental health in the Republic of Georgia: a cross-sectional study
Source: Global Health. 2020 Nov 18;16:111. doi: 10.1186/s12992-020-00641-9 (PMC7672175; doi:10.1186/s12992-020-00641-9)
Supplement: Supplementary file 4 — Additional file 4. Online Annex 4. Detailed results for symptoms of mental disorders, by previous mental health diagnosis status (N = 2024). [file 12992_2020_641_MOESM4_ESM.docx]

**Online Annex 4: Detailed results for symptoms of mental disorders, by previous mental health diagnosis status (N=2,024)**

| **Mental disorder**  (scoring cut-offs) | **No previous mental health diagnosis (N=1722)** | | | | | | | | |  | **Previous mental health diagnosis status (N=302)** | | | | | | | | | | |
| --- | --- | --- | --- | --- | --- | --- | --- | --- | --- | --- | --- | --- | --- | --- | --- | --- | --- | --- | --- | --- | --- |
|  | **Women (N=1495)** | | | |  | **Men (N=227)** | | | |  | **Women (N=260)** | | | | | |  | **Men (N=42)** | | | |
|  | **N** | **%** | **[95% Cl]** | |  | **N** | **%** | **[95% CI]** | |  | **N** | | **%** | | **[95% CI]** | |  | **N** | **%** | **[95% CI]** | |
| **Anxiety symptoms**  **(GAD-7):** |  |  |  |  |  |  |  |  |  |  |  |  | |  | |  |  |  |  |  |  |
| Minimal (0-4) | 611 | 40.87 | [38.40; | 43.38] |  | 114 | 50.22 | [43.71; | 56.72] |  | 56 | 21.54 | | [16.94; | | 26.98] |  | 15 | 35.71 | [22.48; | 51.56] |
| Mild (5-9) | 576 | 38.53 | [36.09; | 41.02] |  | 75 | 33.04 | [27.20; | 39.45] |  | 98 | 37.69 | | [31.98; | | 43.77] |  | 10 | 23.81 | [13.07; | 39.38] |
| Moderate (10-14) | 210 | 14.05 | [12.37; | 15.90] |  | 26 | 11.45 | [7.90; | 16.32] |  | 64 | 24.62 | | [19.74; | | 30.24] |  | 13 | 30.95 | [18.60; | 46.80] |
| Severe (15-21) | 98 | 6.56 | [5.41; | 7.93] |  | 12 | 5.29 | [3.02; | 9.10] |  | 42 | 16.15 | | [12.15; | | 21.17] |  | 4 | 9.52 | [3.51; | 23.33] |
| *Mean anxiety score* |  | *6.28* | *[6.04;* | *6.51]* |  |  | *5.37* | [4.75; | 5.99] |  |  | *8.93* | | *[8.28;* | | *9.59]* |  |  | *7.90* | [6.10; | 9.71] |
| **Depression**  **(PHQ-9):** |  |  |  |  |  |  |  |  |  |  |  |  | |  | |  |  |  |  |  |  |
| Minimal (0-4) | 544 | 36.39 | [33.98; | 38.86] |  | 91 | 40.09 | [33.88; | 46.63] |  | 39 | 15.00 | | [11.14; | | 19.90] |  | 9 | 21.43 | [11.32; | 36.82] |
| Mild (5-9) | 555 | 37.12 | [34.71; | 39.61] |  | 91 | 40.09 | [33.88; | 46.63] |  | 94 | 36.15 | | [30.52; | | 42.20] |  | 14 | 33.33 | [20.52; | 49.20] |
| Moderate (10-14) | 240 | 16.05 | [14.28; | 18.00] |  | 28 | 12.33 | [8.64; | 17.32] |  | 56 | 21.54 | | [16.94; | | 26.98] |  | 8 | 19.05 | [9.62; | 34.22] |
| Moderately severe (15-19) | 103 | 6.89 | [5.71; | 8.29] |  | 12 | 5.29 | [3.02; | 9.10] |  | 40 | 15.38 | | [11.47; | | 20.32] |  | 6 | 14.29 | [6.40; | 28.88] |
| Severe (20-27) | 53 | 3.55 | [2.72; | 4.61] |  | 5 | 2.20 | [0.92; | 5.20] |  | 31 | 11.92 | | [8.50; | | 16.48] |  | 5 | 11.90 | [4.91; | 26.13] |
| *Mean depression score* |  | *7.18* | *[6.91;* | *7.46]* |  |  | *6.26* | [5.60; | 6.93] |  |  | *10.75* | | *[9.97;* | | *11.53]* |  |  | *10.38* | [8.15; | 12.61] |
| **PTSD**  **(ITQ):** |  |  |  |  |  |  |  |  |  |  |  |  | |  | |  |  |  |  |  |  |
| PTSD symptoms | 134 | 8.96 | [7.51; | 10.41] |  | 22 | 9.69 | [5.81; | 13.57] |  | 66 | 25.38 | | [20.06; | | 30.71] |  | 10 | 23.81 | [10.38; | 37.24] |
| **Adjustment Disorder**  **(ADNM-8):** |  |  |  |  |  |  |  |  |  |  |  |  | |  | |  |  |  |  |  |  |
| Adjustment disorder symptoms (18.5-32) | 537 | 35.92 | [33.48; | 38.35] |  | 63 | 27.75 | [21.88; | 33.62] |  | 169 | 65.00 | | [59.16; | | 70.84] |  | 17 | 40.48 | [24.99; | 55.96] |
| *Mean adjustment disorder score* |  | *17.04* | *[16.73;* | *17.35]* |  |  | *15.49* | *[14.74;* | *16.25]* |  |  | *21.76* | | *[21.00;* | | *22.52]* |  |  | *18.90* | [16.82; | 20.99] |
